# Therapeutic Potential of the Gut Microbiota in the Management of Sepsis

**DOI:** 10.1186/s13054-020-2780-3

**Published:** 2020-03-24

**Authors:** Matteo Bassetti, Alessandra Bandera, Andrea Gori

**Affiliations:** 1 Department of Health Sciences, University of Genoa and Ospedale Policlinico Martino-IST, IRCCS, Genoa, Italy; 2 Infectious Disease Unit, Department of Internal Medicine, Fondazione IRCCS Ca’ Granda, Ospedale Maggiore Policlinico, Milan, Italy; 3 Department of Pathophysiology and Transplantation, University of Milano, Milan, Italy

## Abstract

This article is one of ten reviews selected from the Annual Update in Intensive Care and Emergency Medicine 2020. Other selected articles can be found online at https://www.biomedcentral.com/collections/annualupdate2020. Further information about the Annual Update in Intensive Care and Emergency Medicine is available from http://www.springer.com/series/8901.

## Introduction

During the last 20 years, the fields of microbiology and infectious diseases have faced a paradigm shift thanks to the discovery of the complex interactions between the host, its immune system, its microbiome, and various pathogens. In fact, the development of various techniques, such as metagenomics, metatranscriptomics, metaproteomics, and metabolomics, has let scientists discover the inner structure of human genetic composition. The human microbiome has been defined as the collective genome of millions of bacteria, viruses, and fungi that exists on every human host. It plays an elegant mutualistic relationship with the human host from birth [1]. Specifically, the human gastrointestinal tract contains trillions of bacteria that compose a complex ecosystem known as the intestinal microbiota that has relevant implications in human health and disease, especially in the hospital setting [2]. Resident microbiota can outcompete pathogens for space, metabolites and nutrients, and can inhibit pathogens with the calibration of the host immune response. Perturbation of these mechanisms is a common starting point for infection, with antibiotic therapy representing the most common cause of microbiome dysregulation [3].

The interaction between sepsis and the microbiome has been defined as an “incompletely understood bi-directional relationship.” Some evidence has shown that a diverse and balanced gut microbiota is able to enhance host immunity to both enteric and systemic pathogens and that disturbance of this balance potentially leads to increased susceptibility of sepsis. On the other hand, other studies have shown that the composition of the intestinal microbiota is severely affected by sepsis and its treatment, but the clinical consequences of these disturbances need to be further investigated. In this chapter, we provide an overview of the mechanisms through which gut microbiota can contribute to both susceptibility and outcome of sepsis. We will then describe potential therapeutic effects of interventions on the gut microbiome in the setting of septic and critically ill patients.

## Mechanisms of Dysbiosis in Sepsis

During recent years, resident gut microbial flora has been identified as a key factor in a broad range of functions, such as food digestion, hormone production, and immune system development. Moreover, it has been demonstrated that a condition of disturbance of the gut microbiota, also termed “dysbiosis,” can definitely influence host susceptibility to infections.

In general, the gut microbiota consists of three domains of life: bacteria, archaea, and eukarya. The human gut microbiota has a large variety of bacterial species—around 200 dominant species and 1000 non-dominant species—and they vary across individuals. The diversity within an individual’s microbiota is known as alpha diversity, whereas different composition between individuals is called beta diversity. Four phyla represent most of the microbiota members: *Bacteroidetes*, *Firmicutes*, *Actinobacteria*, and *Proteobacteria*, the former and the latter accounting for more than 90% of the bacterial population of the colon. The bacteroidetes phylum is composed of Gram-negative, rod-shaped bacteria that digest complex polysaccharides with the release of volatile short-chain fatty acids that regulate intestinal epithelial cell growth as well as differentiation and stimulation of the immune system. The *Firmicutes* phylum is composed mainly of Gram-positive bacteria that can form endospores (*Clostridia* class). These bacteria release butyrate, promoting intestinal epithelial health and inducing colonic T regulatory cells. However, these phyla contain clinically relevant members such as *Bacteroides fragilis*, *Clostridium perfringens*, *Clostridium difficile*, *Enterococcus* spp., and *Streptococcus* spp. that can cause sepsis and fatal outcome during intestinal dysbiosis [2]. As the composition of the gut microbiota is specific for each person, dysbiosis can be interpreted as a relative change in the composition of an individual’s commensal microbiota compared with others in the community, which can be loss of beneficial microbiota, increased pathogenic microbiota or decreased microbiota variety. Several mechanisms presenting during gut barrier dysfunction can be considered both a result and a cause of sepsis development: the increased permeability of gut mucosa, tissue edema, reduced perfusion, dysregulation of tissue coagulation, shift in the gut microbiome, apoptotic damage to the mucosal epithelia, and bacterial translocation. Gut mucosal perfusion is reduced during sepsis, which produces destruction of the mucosal barrier and increased permeability [4]. Transmigration of bacteria and endotoxin can induce relevant systemic effects, inducing an immune response in the local gut-associated lymphoid tissue (GALT), which in turn activates Toll-like receptor (TLR)4 and priming neutrophils, causing remote lung injury, explaining the appearance of acute respiratory distress syndrome (ARDS) during sepsis [5]. The dysregulation between diverse resident bacterial populations in the gut can lead to a “pathobiome” that finally dysregulates the immune system [6] (Box 1). Indeed, in critically ill patients, hypoxic injury, disrupted epithelial permeability, altered gut motility, and treatment with vasopressors, parenteral nutrition, and opioids facilitate the expansion of pathobionts, including multidrug-resistant (MDR) bacteria [8]. Commonly, the gut microbiome of septic intensive care unit (ICU) patients demonstrates a loss of microbial richness and diversity, dominance of a single taxon (often a potential pathogen), and loss of site specificity with isolation of the same organism at multiple sites [9]. The duration of ICU dysbiosis, the clinical impact of dysbiosis, and phenotypes of critically ill patients more prone to develop it are all aspects that need to be clarified.

**Box 1 Glossary of terms**

**Table:** 

Microbiome	Collective genome of millions of bacteria, viruses, and fungi that exists on every human host
Microbiota	The totality of microbial genomes in a definite host or organ
Pathobiome	The dysregulation between diverse resident bacterial populations in the gut
Dysbiosis	Condition of disturbance of gut microbiota
Metagenomics	The study of the collective genomes of a given community of microorganisms
Metabolomics	The study of the total small metabolites present in a given environment
Alpha diversity	The diversity within an individual’s microbiota
Beta diversity	Different composition of microbiota between individuals
Prebiotics	Nutrients that favor the growth and predominance of beneficial microbes and their inherent functions
Probiotics	Live microorganisms which, when administered in adequate amounts, confer a health benefit on the host
Synbiotics	Combination of probiotics and prebiotics

Adapted from [7]

## Dysbiosis as a Potential Risk Factor for Sepsis

It is generally assumed that sepsis mortality is due to an immunologic disorder, where the causative pathogen is considered irrelevant once the deregulated immune response has begun [10]. As a healthy gut microbiota has been demonstrated to have protective effects on the host and to prevent colonization with MDR bacteria, several researchers have hypothesized that shifts in microbiota composition potentially predispose patients to a state of immunosuppression and thus increase the risk of sepsis.

In an animal model of mice fed with an obesogenic Western diet, a diet high in fat and sucrose and low in fiber, it has been recently demonstrated that they become susceptible to lethal sepsis with multiple organ damage after exposure to antibiotics and an otherwise-recoverable sterile surgical injury. Analysis of the gut microbiota in this model demonstrated that the Western diet alone led to loss of *Bacteroidetes*, increased *Proteobacteria*, and had evidence of antibiotic resistance development even before antibiotics were administered. In this elegant work, it was clearly shown how the selective pressures of diet, antibiotic exposure, and surgical injury can converge on the microbiome, resulting in lethal sepsis and organ damage without the introduction of an exogenous pathogen [11].

A similar recent study conducted by Napier and colleagues, while confirming the effect of Western diet on disease state and outcomes of a lipopolysaccharide (LPS)-driven sepsis model, found that this relationship was independent of the microbiome. Indeed, they demonstrated that Western diet-fed mice had higher baseline inflammation and signs of sepsis-associated immunoparalysis compared with mice fed with standard fiber-rich chow. Western diet mice also had an increased frequency of neutrophils, some with an “aged” phenotype, in the blood during sepsis compared with standard fiber-rich mice. Importantly, they found that the Western diet-dependent increase in sepsis severity and higher mortality was independent of the microbiome, suggesting that the diet may be directly regulating the innate immune system through an unknown mechanism [12].

This preclinical observation has been confirmed by some limited clinical studies in which patients who developed sepsis showed an altered microbiota pattern at baseline. In a recent study, differences in the gut microbiota and plasma LPS level were evaluated in 32 patients who underwent splenectomy and 42 healthy individuals. The splenectomy group was divided into three subgroups according to the length of their postoperative time. Significant differences were observed in gut microbiota composition measured by 16s rRNA gene sequencing with regard to the relative bacterial abundances of 2 phyla, 7 families, and 15 genera. The LPS level was significantly higher in the splenectomy group than in healthy controls and was negatively associated with five bacterial families with low abundance in the splenectomy group. Interestingly, the degree of gut microbiota alteration increased with the length of the postoperative time [13]. Similarly, a seminal study showed that patients undergoing allogeneic bone marrow transplantation who developed antibiotic-induced dysbiosis had a five- to ninefold increased risk of bloodstream infection and sepsis [14]. These observations were confirmed by a retrospective cohort study including over 10,000 elderly patients in the United States and showing that dysbiosis was associated with a more than threefold increased incidence of a subsequent hospitalization for sepsis [15]. Expanding on these findings, Baggs et al. recently showed that exposure to longer durations of antibiotics, additional classes of antibiotics and broader-spectrum antibiotics during hospitalization were each associated with dose-dependent increases in the risk of subsequent sepsis. This association was not found for other causes of hospital readmissions, suggesting that the association between antibiotic exposure and subsequent sepsis is related to microbiome depletion, not to severity of illness [16].

Accumulating evidences thus indicate that gut microbiota disruption may increase the risk of sepsis; future innovations focused on restoring or protecting the gut microbiota from disruption might become a possible approach for preventing sepsis, especially in fragile populations.

## The Gut Microbiota as a Predictor of Clinical Outcome in Sepsis

The transition of a microbiome into a pathobiome has also been hypothesized to be a driver of severe outcome and mortality from sepsis, at least in part by the ability of invading bacteria to act as antigens and thus modulate the host immune response.

In animal models, the effect of the gut microbiome on sepsis outcome has been clearly demonstrated by different studies. In a well-designed recent study, sepsis evolution was analyzed in genetically identical, age- and sex-matched mice obtained from different vendors and subjected to cecal ligation and puncture (CLP), the most frequently used model of sepsis [17]. Beta diversity of the microbiome measured from feces of mice coming from two different laboratories demonstrated significant differences and, more importantly, mice from the first lab had significantly higher mortality following CLP, as compared to mice from the second lab (90% vs. 53%). Differences were also found in immune phenotypes in splenic or Peyer’s patch lymphocytes. To verify if the differences in the microbiome were responsible for the different outcomes, mice were co-housed for 3 weeks, after which they assumed a similar microbiota composition. Interestingly, co-housed mice had similar survival regardless of their vendor of origin and differences in immune phenotype disappeared. This elegant experiment clearly shows that the microbiome plays a crucial role in survival from and in the host immune response to sepsis, representing a potential target for therapeutic intervention.

Clinical studies also confirmed the observation that outcome of sepsis could be influenced by gut microbiota disruption. In the ICU setting, Shimizu et al. quantitatively measured changes in gut microbiota in patients with systemic inflammatory response syndrome (SIRS). These patients had 100–10,000 times fewer total anaerobes, including *Bifidobacterium* and *Lactobacillus*, and 100 times more *Staphylococcus* bacteria compared with healthy volunteers. An important finding of this study was that the dominant factors associated with mortality and septic complications were the numbers of total obligate anaerobes [6]. To evaluate the effect of dynamics of the gut microbiome, a single-center study prospectively analyzed 12 ICU patients and showed that changes in the gut microbiota can be associated with patient prognosis [18]. Indeed, the proportions of *Bacteroidetes* and *Firmicutes* significantly changed during the stay in the ICU, and “extreme changes” in the *Bacteroides/Firmicutes* ratio were observed in almost all the patients with a poor prognosis, suggesting a correlation between alteration in gut microbiota composition and sepsis outcome [18].

The gut has been also hypothesized to be “the motor” of multiple organ dysfunction syndrome (MODS), as reviewed by Klingensmith and Coopersmith [19]. Indeed, evidence from models of murine sepsis and from human patients with ARDS has shown that the lung microbiota is enriched by bacteria translocating from the gut. Importantly, the presence of these bacteria, such as *Bacteroides* spp, is associated with the grade of systemic and local inflammation [20]. Moreover, preliminary studies performed in mice and in patients dying from sepsis suggest that microbial translocation from the gut can be related to neuro-inflammation in sepsis [21]. All these observations provide evidence that dysbiosis observed during sepsis could potentially contribute to worsening inflammation and consequently severe clinical outcome. However, well-designed human clinical studies are still needed as our current knowledge of the consequences of ICU-related dysbiosis in clinical practice is limited.

## Modulation of the Microbiota as Potential Therapeutic Immunonutrition

Probiotics are considered as living microorganisms, which, in adequate amounts, can induce health benefits to the human host. Among them, the genera *Lactobacillus* and *Bifidobacterium* are the most widely used. Probiotics have been increasingly applied and studied in different clinical applications. Probiotics have been hypothesized to reduce the risk of disease through competition for binding locus and nutrients with pathogens, producing bacteriocins to kill pathogens, synthesizing IgA to support immune responses and reducing inflammation. Prebiotics are defined as a non-digestible food ingredient that beneficially impacts the host by stimulating the growth and/or activity of a limited number of bacterial species in the gut. Synbiotics are composed of probiotics and prebiotics.

In the context of sepsis models and ICU patients, probiotics have been studied and evaluated in terms of sepsis evolution and subsequent outcome. A study by Chen and coauthors reported that prophylactic administration of a probiotic bacterial species in a septic mouse model effectively reduced mortality [22]. More recently, a study conducted on a model of septic mice specifically demonstrated that after the onset of sepsis, there was an appearance of opportunistic gut pathogens such as *Staphylococcaceae* and *Enterococcaceae* and a disappearance of beneficial *Prevotellaceae* [23]. Relative abundance of potentially pathogenic commensals was associated with more severe immune responses during sepsis, demonstrated by higher peripheral pro-inflammatory cytokine levels, gut epithelial cell apoptosis, and disruption of tight junctions. Interestingly, in animals pre-treated with *Lactobacillus rhamnosus* GG, opportunistic pathogens decreased or even disappeared, while beneficial bacteria, such as *Verrucomicrobiaceae*, increased, promoting inhibition of gut epithelial cell apoptosis and tight junction formation. Moreover, in a novel *in vitro* gut model to study *Candida* pathogenicity, the introduction of a microbiota of antagonistic lactobacilli emerged as a significant factor for protection against *C. albicans*-induced necrotic damage, with a time-, dose-, and species-dependent protective effect of probiotics against *C. albicans*-induced cytotoxicity [24].

Use of prebiotics/probiotics/synbiotics in clinical ICU studies has been evaluated in many small studies in different populations (summarized in Table 1): (1) to prevent infections, especially in the context of postoperative and mechanically ventilated patients; (2) to improve outcome of sepsis; (3) to restore gut commensals after sepsis to reduce late infections and subsequent mortality.

**Table 1:** Immunonutrition in critically ill patients: clinical settings, outcomes, and research gaps

Clinical settings	Products	Outcomes	Research gaps
Mechanically ventilated patients	Probiotics Synbiotics	Incidence of VAP	Choice of probiotics/synbiotics; dosing and route of administration VAP definition Adverse effects evaluation Paucity of data on the effect on MDR colonization
Elective surgery/ trauma	Probiotics Synbiotics	Incidence of postoperative/post-traumatic infections	Paucity of data on microbiota before surgery Choice of probiotics/synbiotics; dosing and route of administration Adverse effects evaluation
Pre-term infants	Probiotics Synbiotics	Incidence of sepsis Incidence of necrotizing enterocolitis	Impact of type of feeding (mother’s milk, donor milk, formula) Evaluation of impact of human milk oligosaccharides on microbiome Choice of single probiotic strain versus multiple strains To address the risk of probiotic-related sepsis and transmission of antibiotic resistance To address the risk of cross-colonization or cross-contamination
ICU patients	Probiotics Synbiotics	Incidence of new infections Mortality ICU length stay	Choice of probiotics/synbiotics; dosing and route of administration To address short- and long-term effects on gut microbiome To address candidate populations and timing of administration

*VAP* ventilator-associated pneumonia, *MDR* multidrug resistant, *ICU* intensive care unit

Administration of probiotics and synbiotics had been demonstrated to reduce infectious complications, and meta-analyses suggest that probiotics are safe and effective at preventing infection in both postoperative and mechanically ventilated patients [25, 26]. However, various concerns have been raised regarding the type and optimal dose of probiotic therapy, as well as the small size of the individual studies. Morrow et al., in the most rigorous study, reported that the incidence of ventilator-associated pneumonia (VAP) in patients treated with *L. rhamnosus* GG was significantly lower than in controls (19.1% vs. 40.0%) in 138 ICU patients. Moreover, probiotic administration significantly reduced oropharyngeal and gastric colonization by pathogenic species [27]. However, other clinical reports showed no significant difference in the occurrence of VAP in the ICU [28]. In a recent randomized controlled study, the effect of prophylactic synbiotics on gut microbiota and on the incidence of infectious complications including enteritis, VAP, and bacteremia was evaluated in mechanically ventilated patients with sepsis. Seventy-two patients completed the trial, of whom 35 patients received synbiotics and 37 patients did not. In the synbiotics group, the incidence of enteritis and the incidence of VAP were significantly lower compared to controls. The incidence of bacteremia and mortality, however, did not differ significantly between the two groups [29]. Currently, we are waiting for the results of a large randomized placebo-controlled study [30] aimed to determine the effect of *L. rhamnosus* GG on the incidence of VAP and other clinically important outcomes (*C. difficile* infection, secondary infections, diarrhea) in critically ill mechanically ventilated patients (Clinicaltrials.gov Identifier: NCT02462590).

Several studies have assessed the role of probiotics in other populations, such as pre-term and underweight children, finding no differences in sepsis incidence and mortality, indicating that the potential effects of microbiota restoration are not uniformly conserved across populations and settings [31, 32]. Interestingly, a recent randomized, double-blind, placebo-controlled trial testing an oral synbiotic preparation (*Lactobacillus plantarum* plus a fructooligosaccharide) in healthy, termneonates in India was interrupted early because of a reduction of 40% in death and sepsis in the treatment arm [33].

The last frontier in the context of immunonutrition is the development of next-generation probiotics able to selectively inhibit specific pathogens, such as *C. difficile* and MDR bacteria, in order to administrate a target population that would support colonization resistance and prevent infections and sepsis [34].

### Fecal Microbiota Transplantation

Fecal microbiota transplantation (FMT) consists of administering fecal material from a healthy donor into the intestinal tract of a patient with an altered gut microbiota to restore its functions. Clinician interest in this treatment was renewed in 2013 with publication of the results of a randomized controlled trial showing the substantial superiority of FMT over standard care in the treatment of recurrent *C. difficile* infections [35]. Based on the absolute number of introduced bacteria, FMT is thought to be the most powerful immunomodulatory tool. In animal models, FMT alone is capable of restoring bacterial communities in cecal crypts, which act as a reservoir of commensal bacteria to restore the intestinal epithelium. Crypts are also crucial in protecting intestinal stem cells and in preservation of immunological pathways by enhancing the expression of nod-like and Toll-like receptors. Depletion of commensal organisms in crypts enhances pathogen proliferation, which can result in severe inflammation and disruption of homeostasis. Another potential advantage of FMT is that, along with the transfer of bacterial communities, other products (short-chain fatty acids, bile acids, eukaryotic, and prokaryotic viruses) are introduced to the intestinal ecosystem, leading to a complete restoration of homeostasis [36].

The rationale for use of FMT in critical illness is fascinating and promising. However, its application in clinical practice among ICU patients is unexplored. We believe that FMT can have a potential role in critical patients in two directions: (1) restoration of ICU-associated dysbiosis and (2) implementation of gut decolonization of MDR organisms. In fact, the introduction of a high burden of commensal bacteria may reverse resistant pathobiont dominance and even decrease the antibiotic resistance genes present in the microbiome (resistome) [37]. However, only five cases have been described in which FMT has been employed to address disruption of the microbiota in the ICU. All these cases showed that treatment with FMT led to a successful reversal of dysbiosis, with subsequent improvement in outcome. In addition, some cases noted a steep decrease in inflammatory mediators and normalized Th1/Th2 and Th1/Th17 ratios following FMT. Apart from difficulties with extrapolating the data derived from these case reports to the general ICU population, we are far from obtaining conclusive evidence that restoration of dysbiosis by FMT in critical illness is beneficial. However, given the promising results of FMT learned from *C. difficile* treatment experience, clinical trials are needed to implement a microbiota-targeted approach.

Colonization with MDR bacteria is a leading cause of sepsis complications especially among vulnerable ICU patients [38]. The use of FMT for this purpose has been evaluated in different case series, retrospective and prospective studies, highlighting that this approach can be feasible safe and effective [39]. Results cannot be easily analyzed because of the high risk of bias in smaller studies, but in a recent review that considered only studies with low and moderate risk of bias, an eradication rate between 37.5% and 87.5% was described [40]. However, results of different studies cannot be conclusive because of different patient populations (with the most commonly organisms isolated pre-FMT being carbapenem-resistant *Enterobacteriaceae* [CRE], vancomycin-resistant *Enterococci* [VRE], and extended-spectrum β-lactamase [ESBL]-producing bacteria, and also *Pseudomonas*, methicillin-resistant *S. aureus* [MRSA], and *Acinetobacter*, and differences in route of administration, choice of donors, and length of follow-up [39]. Recently, a randomized controlled trial has been completed showing that patients given nonabsorbable oral antibiotics followed by FMT had a slight decrease in ESBL and CRE colonization compared with control patients, although without reaching statistical significance. The unfavorable results are potentially due to the study design (two different routes of FMT in the interventional group and contemporary antibiotic administration may have influenced carriage in the interventional group) and early trial termination [41]. However, it is important to note that so far none of the published studies has been conducted in ICU patients. Until now, only one pilot study is ongoing among ICU patients with a prevision of enrollment of 10 mechanically ventilated patients with MDR colonization (Clinicaltrials.gov Identifier: NCT03350178).

Various concerns specific to ICU patients have been raised in addition to other unanswered questions regarding FMT itself (e.g., transmission of pathogens, dose, route, and long-term safety), as well as several practical aspects that need to be investigated. First, we do not know which candidate population of septic patients is best and what the correct timing of FMT administration is in relation to antibiotic use because of the risk of nullifying the effects of transplantation.

A microbiota suspension as a fecal filtrate transfer (FFT) seems to maintain the ability to stimulate host responses via pattern recognition receptors enabling ecologic niches to be modified for outgrowth of existing beneficial bacteria or even successful novel colonization [42]. This characteristic, together with a possibility to create a capsule, can increase the chances of successful FMT application even during antibiotic treatment, reducing also the potential risk of instillation of large bacterial burdens among immunocompromised patients.

Furthermore, more experience is crucial to evaluate what is the best route of administration (colonoscopy or enema vs nasogastric tract) and use of autologous vs heterologous transplantation. Colonoscopy or enema are the most commonly used methods of stool delivery. A randomized study found that FMT using the nasogastric tract was less effective than colonoscopy [43]. Expert opinion tends to favor colonoscopy because of its ability to visualize the entire colon and to deliver larger amounts of stool near the affected pathological segment of the bowel [44]. Moreover, non-inferiority of capsule use over colonoscopy was demonstrated in a randomized study [45].

Finally, the use of autologous vs heterologous FMT needs to be clarified because autologous FMT can have a higher potential application in the ICU setting among patients receiving solid or hematopoietic transplant in an attempt to prevent infections after a period of dysbiosis.

In conclusion, we believe that the potential benefits from FMT (regarding the control of MDR bacteria and *C. difficile* infection) justify the investigation of this promising approach in ICU patients.

## Conclusion and Future Perspectives

Despite the impressive achievement that has been made in knowledge of the microbiome, there is still a huge gap about the microorganisms that reside outside the gut and interactions of bacteria with viruses, archeae, helminths, fungi, and protozoa, which influence each other and in turn regulate the host. In the context of critically ill septic patients, we need large human cohort studies that document microbiota composition, prior to, during and after an episode of sepsis in order to identify protective commensals and microbiota potentially associated with increased susceptibility and worse outcome.

At the same time, new treatment opportunities are gaining space in clinical practice, including the addition of a probiotic, or by tailoring microbiome therapy and selecting specific commensal repletion that could target a specific infectious disease. In this setting, human studies and randomized clinical trials are challenging but still fundamental in order to translate basic research into innovative paradigms.

## Data Availability

Not applicable.
